# Incidence and determinants of diabetic ketoacidosis among people with diabetes in Woldiya comprehensive specialized hospital, Ethiopia: a retrospective cohort study

**DOI:** 10.1186/s12902-024-01552-1

**Published:** 2024-03-11

**Authors:** Beyene Zewdu, Tefera Belachew, Kemal Ahmed, Lehulu Tilahun, Kirubel Dagnaw

**Affiliations:** 1 Department of Nursing, Raya Kobo Primary Hospital, Kobo, Ethiopia; 2 College of Medicine and Health Sciences, Jimma University, Jimma, Ethiopia; 3 School of Public Health, College of Medicine and Health Sciences, Wollo University, Dessie, Ethiopia; 4 Department of Emergency and Critical Care Nursing, College of Medicine and Health Sciences, Bahir Dar University, Bahir Dar, Ethiopia; 5 Department of Comprehensive Nursing, College of Medicine and Health Sciences, Wollo University, Dessie, Ethiopia

**Keywords:** Incidence rate, Determinants, Diabetic ketoacidosis, Ethiopia

## Abstract

**Introduction:**

Diabetic ketoacidosis is an acute complication of diabetes mellitus that is characterised by hyperglycemia, acidosis, and ketonuria. Diabetes is the most challenging public health problem in the twenty-first century for both developed and developing countries.

**Objective:**

To assess the incidence of Diabetic ketoacidosis and its determinants among adult people with diabetes at an Ethiopian Hospital.

**Method:**

An institution-based retrospective cohort study was conducted among 390 adult people with diabetes attending services at Wolida Comprehensive Specialized Hospital. The consecutive sampling method was used to select study participant charts. Data were collected using a checklist prepared from different literature. The data were entered into EPI data version 4.6.0.5 and exported to STATA version 14.0 for further analysis. The Wiebull model was the best fitted model that was selected using the log-likelihood ratio method and the Akakian information criterion. Hazard ratios with their 95% confidence interval and *p*-value were computed.

**Result:**

From the total 405 charts reviewed, 390 adult charts were included for analysis. A total of 121 DKA occurred from 5471 person–months of observation. The overall incidence rate of diabetic ketoacidosis was found to be 2.2 per 100 person-months (95% CI: 1.8- 2.6). Being urban dweller (AHR: 0.59, 95% CI: 0.36–0.99), having no family history of DM (AHR: 0.55, 95%CI: 0.31—0.97), presence of infection (AHR: 2.60, 95%CI = 1.06–6.39), having of any comorbidities (AHR: 4.31, 95% CI: 1.70–10.90), and having poor glycemic control (AHR: 7.45, 95% CI: 3.84–14.47) were significant determinants.

**Conclusion and recommendations:**

The overall incidence of diabetic ketoacidosis in study area was relatively high. Poor glycemic control, the presence of infection, and comorbidity were determinants of diabetic ketoacidosis. There is a need to have close follow-up of people with diabetes who have comorbidity, infection, and poor glycemic control.

**Supplementary Information:**

The online version contains supplementary material available at 10.1186/s12902-024-01552-1.

## Introduction

Diabetes mellitus (DM) encompasses a group of metabolic disorders that share a common phenotype of hyperglycemia. Generally, diabetes mellitus is classified into two types: Namely, type one diabetes mellitus (T1D) results from a deficiency of insulin because of B-cell distraction, and type two diabetes mellitus (T2D), which relates to insulin action [1, 2]. Diabetes was considered a disease of developed countries and wealthy people. But, currently, it affects all populations in the world, particularly in resource-limited countries [3].

Diabetes mellitus has acute and chronic complications. Diabetic ketoacidosis (DKA) is one of the acute complications of DM. DKA is seen primarily in individuals with T1D. DKA results from insulin deficiency due to inadequate insulin administration, infection, surgery, or drugs [4]. Insulin deficiency and effects of counter-regulatory hormones like cortisol, glucagon, epinephrine, etc. become devastating because of decrement in insulin availability and increment in insulin demand [5, 6]. Clinical features of DKA include anorexia, nausea, vomiting, polyuria, thirst, abdominal pain, and altered mental status. Its managements are intravenous administration of fluid, soliciting underline precipitating factors, potassium replacement and administering insulin [2, 5, 7, 8].

Diabetes is most challenging public health problem in twenty-first century [9]. Globally, in 2019, total number of people living with DM among adults was estimated to be about 463 million (9.3%). One in two (50%) of people living with diabetes do not know that they have diabetes [10]. Unless measures taken the total number of adults afflicted by DM will increase from 463 million in 2019 through 578 million in 2030 to 700 million in 2045 [11]. In 2018, prevalence of DM in the USA among adults aged greater than 18 years was estimated to be 34.1 million. In the USA, in 2016, the crude rates of emergency visits and hospitalizations of DKA per 1000 adults were estimated to be 8.8 and 8.1, respectively [12].

Africa is a continent with a high prevalence of DM. In 2019, the number of adults afflicted by DM and diabetic-related deaths was about 19 million and 366,200, respectively [11, 13]. In sub-Saharan Africa, the prevalence of DM dramatically increased in line with other non-communicable disease [14].

In Ethiopia, in 2019, prevalence of DM and DM-related deaths among adults with diabetes mellitus was estimated to be about 3.2% and 23,157, respectively [13]. As prevalence of diabetes continues to increase, diabetes complications continued to be a public health concern [15]. A systematic review of the study undertaken in 2017 indicates that the incidence and prevalence of DKA in T1D were about 0–56/1000 PYs and 0–128/1000 PYs, respectively [16]. In Ethiopia, incidence of DKA in adult DM patients is unknown, but a study in Hawassa revealed that the prevalence of DKA was about 40% [17].

DKA is a serious, life-threatening acute complication of DM. The mortality rate varies from 2 to 5 percent and from 6 to 24 percent for developed and developing countries, respectively [17]. In 2019, the mortality rate of DKA was reported to be about 23.6% [18]. The economic burden of DM can be direct, including direct medical expenditure for prevention and treatment of DM and DM complications, and indirect, which is associated with productivity loss and premature death [19]. Treatment of DKA requires a large number of resources, accounting for an estimated total cost of $2.4 billion annually [20]. The average cost of treating an episode of diabetic ketoacidosis in an adult patient was estimated to be £2064 [21]. In 2015, the estimated cost of DM in sub-Saharan Africa was about $19.45 billion [22]. Age and urban residence were identified as sociodemographic predictors of DKA [17, 23, 24]. Besides the above, T1D, the presence of comorbidity, infection prior to the onset of DKA, poor glycemic control, and medication discontinuation are associated with DKA [25–27].

To prevent morbidity and mortality attributed to non-communicable diseases (NCDs), Ethiopia set up a strategic action plan for prevention and control of NCDs with four priority areas. These are, strengthen the national response through policy, governance, and leadership, health promotion, and disease prevention targeting behavioural risk factors [28].

Although there were many adult people with diabetes in Ethiopia, there has been a paucity of evidence on the incidence of DKA and its causes so far. Most previous studies were conducted on childhood people with diabetes and most of the studies were conducted using case–control studies, so the representativeness and generalizability of the findings were limited because of the introduction of different biases. Therefore, the purposes of this study are to assess the incidence of DKA and identify determinants of DKA among adult people with diabetes using a retrospective cohort study.

## Methods and materials

### Study design

An institution-based retrospective cohort study design was used. The study was conducted from April 19 to May 17, 2021, at Wolidya Comprehensive Specialized Hospital, which is located 521 km away from Addis Ababa, the capital city of Ethiopia, and 328 km away from Bahir Dar, the capital city of Amhara Region. Woldiya Comprehensive Specialized Hospital has 250 beds and serves about 3.5 million people in the North Wollo Administrative Zone and neighbouring regions like Afar and Tigray. The Hospital provided chronic care and follow-up services. Currently, the total number of adult people with diabetes attending services at Wolidya Comprehensive Specialized Hospital is 788.

All adult people with diabetes who were on follow up at Wolidya Comprehensive Specialized Hospital were the source population, whereas all consecutively selected records of adult people with diabetes who were enrolled in diabetic follow-up service from January 1, 2016 to January 1, 2021 at Wolidya Comprehensive Specialized Hospital were the study population.

Age greater than or equal to 18 years and people with diabetes who enrolled in diabetic follow up service from January 1, 2016 to January 1, 2021 were illegible for this study. But adults who developed DKA at the first diagnosis of DM were excluded from the study.

### Sample size determination and sampling technique

The sample size was determined by using Epi info version 7 to calculate the minimum required sample size by considering the following assumptions: Level of significance (α) = 5%, power = 80%, and Z a/2 (value at 95% confidence level = 1.96, margin of error (5%), and ratio of unexposed to exposed (1:1) Therefore, the minimum sample size required for attaining statistically significant result was 405 after adding contingencies. This was determined by considering age, presence of infection, and comorbidity as predictors of DKA from previous studies done in Iraq and the North Wollo and Wagihmra zone [27, 29] (Additional file 5).

After medical charts of adults diagnosed with DM were entered into chronic outpatient department (OPD) for follow-up, consecutive sampling technique was applied to select the predetermined sample size (405). To ascertain the final status (develop DKA or be censored), the selected card was followed for maximum of five years.

### Data collection tool and method

The study exclusively used secondary data. Therefore, a data extraction check list was designed from different literature. The checklist contains socio-demographic characteristics, disease-related factors, and treatment related factors. Two data collectors and one supervisor nurse were recruited. From January 1, 2016, to January 1, 2021, different registration formats for adult people with diabetes were reviewed. Data were extracted from the patient’s medical chart, laboratory report, nursing care plan, and treatment chart.

### Data quality control

To ensure data quality, two days of training were provided for data collectors and supervisors on the objective of the study and the procedure they have to follow when obtaining data from medical record charts. Before the actual data collection, a pretest was done on a 5% sample size in Woldiya Comprehensive Specialized Hospital on 22 people with diabetes. Based on the pretest result, certain amendments were made. Strict supervision was provided by the principal investigator and supervisor. On a daily basis, the collected data was checked for completeness, accuracy, and consistency by the principal investigator, and anything that was unclear was corrected and communicated to the data collectors on the next day.

### Data processing and analysis

The data were entered into EPI Data version 4.6.0.5 and exported to STATA version 14.0 for further analysis. The proportional hazard assumption was checked using a log–log plot and Schoenfeld residual. A descriptive statistical analysis (mean, median, and interquartile range) was done to describe the characteristics of the participant. The study participant contribution per person year was calculated by comparing the diagnosis of DM time with the onset of DKA. A descriptive survival statistical analysis (Kaplan–Meier) was used to estimate the time to develop DKA from the diagnosis of DM. The Nelson-Aalen cumulative curve was used to describe the cumulative probability of DKA. For the nested model, the likelihood ratio test was used for model comparison.

For non-nested model information criteria, AIC and BIC were used as model comparisons. Semi-parametric survival models (Cox regression) and parametric survival models (exponential distribution, Weibull distribution, log-normal, and log logistic) were evaluated for fitness to the data set. Model fitness was checked using the Cox-Snell residual test. Weibull model was best fitted model for data. Bi-variable and multivariable Weibull regression analyses were performed to identify determinants of DKA. In bivariable analysis, those variables with a *P* value less than 0.25 were entered into multivariable analysis. Both crude and adjusted hazard ratios were computed with a 95% confidence interval. In multivariable analysis, variables with a *P*-value less than 0.05 were used to declare the statistical significance of the findings in this study.

## Results

### Descriptive characteristics

From the total expected 405 diabetic patient charts, 390 were included in the study, yielding a 96% response rate. Two hundred (51.3%) of the participants were female. The median age of participants was 26 years, with an interquartile range (IQR) of 29–23 years. Two hundred eleven (54.1%) of the participants were orthodox religious followers. Muslims and protestants accounted for 135 (36.4%) and 44 (11.3%), respectively.

Three hundred three (77.7%) of the study participants had T2D. Among 390 adult people with diabetes, charts were reviewed and revealed that almost 33% of participants had comorbidity at baseline. Regarding baseline infection, 54 (45.8%) of adults had a urinary tract infection, whereas 37 (31.4%) had a respiratory tract infection. Whereas, regarding baseline other diabetic complications, 48% of adults had peripheral neuropathy, 2% had diabetic foot ulcers, 8% had ulcers, and 4% had retinopathy. Among the 390 charts reviewed, two hundred 98 (76.9%) of participants used insulin as a treatment for DM. Two hundred thirty-four (60%) of the study participants had community-based health insurance (Table 1).

**Table 1:** Descriptive characteristics of DM patients on follow-up at Woldiya Comprehensive Specialize Hospital from January 2016 to January 2021

Characteristics	Frequency (*n* = 390)	Percent
Age		
18–24	147	37.7
25–30	159	40.8
31–35	21	5.4
Above 35	63	16.1
Sex		
Male	190	48.7
Female	200	51.3
Marital status		
Married	198	50.8
Single	109	27.9
Divorced	76	19.5
Windowed	7	1.8
Residence		
Rural	216	55.4
Urban	174	44.6
Occupational status		
Student	65	16.7
Farmer	77	19.7
Merchant	157	40.3
Government employee	44	11.3
Others	47	12
Religion		
Orthodox	211	54.1
Muslim	135	34.6
Protestant	44	11.3
Infection		
Yes	125	32.05
No	265	67.95
Acute recent illness		
Yes	79	20.3
No	311	79.7
Comorbidity		
Yes	169	43.33
No	225	56.67
Other diabetic complications		
Yes	27	6.92
No	363	93.08
FBSL		
Good	185	47.44
Poor	205	52.56
Type of DM		
T1M	297	76.15
T2M	93	23.85
Type of drug		
Insulin	298	76.91
OHA	62	15.90
Both	30	7.69
Omission of medication		
Yes	121	31
No	269	69
Regular follow –up		
Yes	275	70.5
No	115	29.5
CBHI		
Yes	234	60
No	156	40

### Incidence of DKA

Three hundred ninety (390) adults diagnosed with DM were followed for a maximum of five years, and one hundred twenty-one (31%) developed DKA.

The overall person-month observations were 5471 adult-month observations. The overall incidence rate of DKA in the cohort was 2.2 per 100 person-month observations (95% CI, 1.8–2.6). Particularly, the incidence of DKA in T1D and T2D was 2.4 per 100 person months (95% CI: 1.9–2.9) and 1.6 per 100 person months (95% CI: 1.1–2.4), respectively.

From those who developed DKA, sixty (49.6%) of them were in the age group of 18–24 years, followed by the 25–30 age group, which accounts for forty-eight (39.7%). Regarding age, the incidence rate of DKA among the 18–24 age group was 2.6 per 100 person months of observation (95% CI, 2.0–3.4). For the age group of 25–30 years, the incidence rate of DKA was 2.1 per 100 person months (95% CI, 1.6–2.8).

Among DKA-developed adult people with diabetes, sixty-one (50.4%) were female, and the rest, sixty (49.6%), were male. The incidence of DKA in females and males was 2.2 per 100 person months (95% CI, 1.8–2.8) and 2.1 per 100 person months (95% CI, 1.6–2.8), respectively.

**Fig. 1:**
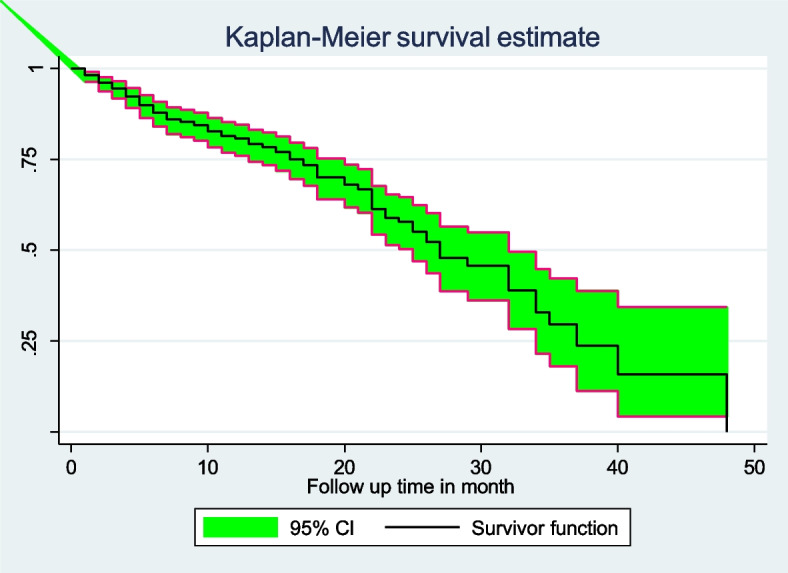
Overall Kaplan–Meier survival curve of adult people with diabetes in Woldiya Comprehensive Specialized Hospital from January 1, 2016 to January 1, 2021

Regarding the time of onset of DKA, 8 (6.61%), 8 (6.61%), 7 (5.79%), 2 (1.65%), and 1 (0.08%) cases of DKA occurred in the first two, four, six, twelve, and twenty-four months of follow-up after diagnosis of DM, respectively. The cumulative survival probabilities were 0.9821, 0.9232, 0.8532, 0.8068, and 0.1580 at 1, 4, 8, 12, and 40 months of DM diagnosis, respectively. It indicated that as time increases, the hazard of diabetic ketoacidosis increases (Fig. 1).

### Determinants of diabetic ketoacidosis

The Weibull model was the best-fit model for the data, with the highest log likelihood of 223.5375 and the lowest AIC of -387.0749 (Additional file 1).

The Cox-Snell residual test indicates that hazard functions against Cox-Snell are parallel to each other. So, the Weibull model is the best-fitted model for data (Additional file 2).

**Fig. 2:**
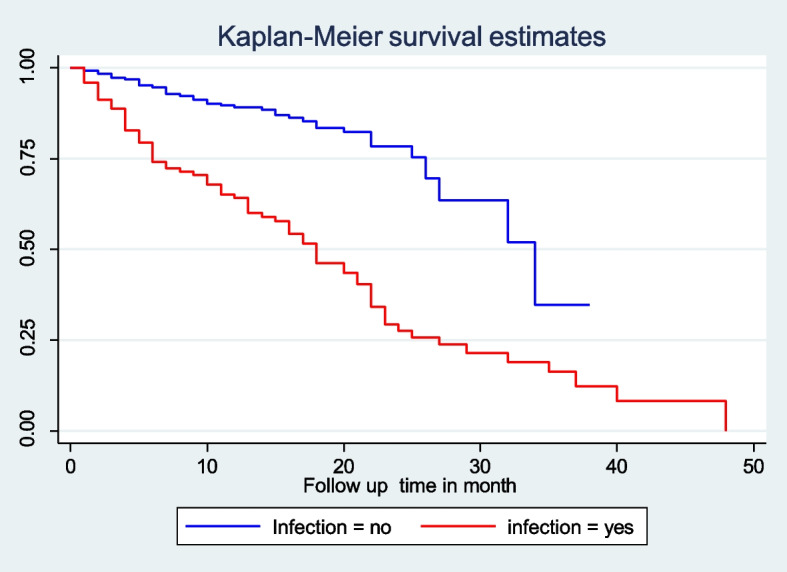
Kaplan-Meiere survival estimate of DKA free survival on infection of people with diabetes in Woldiya Comprehensive Specialized Hospital from January 1, 2016 to January 1, 2021

In this retrospective follow up study, the median survival time of people with diabetes who had infection at baseline was 18 (IQR 27 ± 6) months, while people with diabetes who did not have infection were 34 months (Fig. 2).

Regarding comorbidity, the median survival time of those people with diabetes who had comorbidity was 18 (IQR 29 ± 7) months, while for those people with diabetes who had good glycemic control, it was 40 months (Additional file 3).

Regarding glycemic control, the median and 25th percentile survival times of those people with diabetes who had poor glycemic control were 16 (IQR 23 ± 6) and 6 months, respectively. While the 25 percentile survival time for those people with diabetes who had good glycemic control was 32 months (Additional file 4).

In bivariable Weibull regression, sex (*P* = 0.64), other diabetic complications (*P* = 0.82), and type of drug (*P* = 0.98) have *p* value s > 0.25. So, these variables were not entered into the multivariable Weibull regression. A multivariable Weibull regression analysis revealed that different socio-demographic and disease related variables were the determinants of DKA. These determinants included urban residence (AHR: 0.60, 95% CI: 0.36–0.97), presence of infection (AHR: 2.45, 95% CI: 1.03–5.81), presence of any comorbidity (AHR: 3.47, 95% CI: 1.43–8.41), no family history of DM (AHR: 0.55, 95% CI: 0.31–0.96), and poor glycemic control (AHR: 7.27, 95% CI: 3.80–13.91) (Table 2).

**Table 2:** Bi-variable and multi-variable Weibull regression analysis of determinants of DKA among adult people with diabetes on follow-up at WCSH from January 1, 2016 to January 1, 2021

Characteristics	Survival status		CHR(95% CI)	AHR(95% CI)	*P*
	DKA	Censored			
Family history of DM					
Yes	84	51		1	
No	22	172	0.14(0.08–0.22)	**0.55(0.31–0.96)**	0.036*
Unknown	15	46	0.33(0.19–0.57)	1.23(0.64–2.37)	0.54
Infection					
No	44	221		1	
Yes	77	48	3.79(2.62–5.49)	**2.45(1.03- 5.81**)	0.042*
Acute recent illness					
No	73	238			
Yes	48	31	3.19(2.21–4.59)	0.97(0.63- 1.50)	0.90
Comorbidity					
No	23	198		**1**	
Yes	98	71	6.25(3.97–9.85)	**3.47(1.43 -8.41)**	0.006*
FBSL					
Good	13	203		**1**	
Poor	108	66	11.88(8.92–20.27)	**7.27(3.80–13.91)**	0.000*
Omission of medication					
No	31	238			
Yes	90	31	10.38(6.88–15.65)	3.16(0.64–15.60)	0.16
Regular follow-up					
No	89	26			
Yes	32	243	0.09(0.06–0.14)	1.59(0.31- 8.09)	0.58
CBHI					
No	78	78			
Yes	43	191	0.33(0.23–0.48)	0.78(0.50–1.22)	0.27

^*^Statistically significant. *AHR* Adjusted Hazard Ratio, *CHR* Crude Hazard Ratio

## Discussion

In this study, the incidence rate of DKA in T1D was 28.8 per 100 PYs (95% CI, 22.8–34.8). This finding is higher than the study conducted in Western Australia (32), which was 0.18 per 100 PYs, and in China [30] which was 1.21 per 100 person-years. The possible reason for this discrepancy is sample size and length of follow-up. For instance, a study undertaken in China lasted twelve (12) years. The incidence rate of DKA in T2D patients was 19.2 per 100 person years (PYs) (95% CI, 13.2–28.8). This finding is in contrary to a study done in Western Australia [31], which was 0.013 per 100 person years, and to a study undertaken in the USA, which was 0.17 per 100 PYs. This discrepancy might be due to difference in study design. That is, the study undertaken in Western Australia used a prospective cohort study, but this study used a retrospective cohort study design. The other possible reason for this discrepancy might be socio-economic and socio-cultural differences in health-seeking behavior.The overall incidence rate of DKA in the cohort was 26.4 per 100 person-years (95% CI, 21.6–31.2). On the contrary, this finding is much higher than the study done in Spain [32] which was 0.06 per 100 person-years, and Western Australia [31] which was 0.04 per 100 person-years. The possible reason for this gap is sample size. For example, in Western Australia (*n* = 1724), the other possible reason for this discrepancy is the socio-cultural deference to health-seeking behavior.

Regarding residence, urban dwellers had a lower risk (40%) of DKA as compared to rural dwellers. This study is in line with a study conducted in Nigeria [23], and Hawassa Comprehensive Specialized Hospitall [17]. On the contrary, a study done in Australia and New Zealand [33, 34] revealed that rural dwellers were at higher risk of DKA than urban dwellers. The possible reason for this discrepancy is difference in sample size and statistical analysis. For example, a study undertaken in Australia and New Zealand used logistic regression, while this study used Weibull regression.

Regarding family history, those people with diabetes with no family history decreased by 45% relative to those with diabetes who have a family history of DM. This finding is contrary to a study done in other parts of Ethiopia, like western Ethiopia [35], and Dilla University Referral Hospital [36]. This discrepancy might be due to a difference in study design. That is, a study conducted at Dilla University Referral Hospital used cross-sectional study design, while this study used retrospective cohort study design. The other possible justification was sampling technique. For example, in a study done at Dilla University Referral Hospital, participants were selected by systematic random sampling, whereas in this study, samples were selected by the consecutive sampling method.

Adult people with diabetes with a superimposed infection were 2.45 times more likely to develop DKA than those who did not have an infection at baseline. This study is in accord with studies done in South Africa [25] and Dilla University Referral Hospital [36]. This might be due to the fact that infection causes the body to produce of hormones like adrenaline and cortisol, which hinder the effect of insulin, or infection causes the body to release cytokine and inflammation that end up with disturbance of insulin action (insulin resistance).

In this study, the hazard of DKA among DM patients with any comorbidity was 3.47 times higher than that of those without comorbidity. The findings of this study are in line with those of studies conducted in Jimma Medical centre [26], Hawassa Comprehensive Specialized Hospital [17], and Waghimra and North wollo Public Facilities [27]. This might be because comorbidities like hypertension, cardiovascular disease, chronic obstructive pulmonary disease (COPD), asthma, surgery, trauma, psychiatric disorders, etc. may cause either dehydration or increase disturbance of the normal function of hormones, particularly insulin.

People with diabetes with poor glycemic control were 7.27 times more likely to develop DKA than those with good glycemic control. This finding is consistent with studies done at Debre Markos Referral Hospita [37] and Ayider Referral Hospital [38]. This might be because poor glycemic control is one of the markers of DKA.

In this study, sex, age, marital status, type of DM, type of drug, medication discontinuation, irregular follow-up, and acute recent illness were not significantly associated. This finding is not in agreement with the findings of the study undertaken in North Wollo and Waghimra public facilities, Debre Markos Referral Hospital, Hawassa Comprehensive Specialized Hospital, Dilla University Referral Hospital, and Iraq [17, 22, 24, 36, 37]. This discrepancy might be due to differences in study design and study settings. For example, a study undertaken in Debre-Markos Referral Hospital [37] and Waghimra and North Wollo Public Facilities [22] used an unmatched case–control study design. Likewise, a cross-sectional study design was applied in Hawassa Comprehensive Specialized Hospital [17], Dilla University Referral Hospital [36], and Iraq [25].

## Limitations and strengths of the study

### Strength

Data were collected by nurses trained in non-communicable diseases. Some variables that were not previously traced were included.

### Limitations

Since the study uses secondary data, some important variables were missed, like anthropometry measurements. Misclassification bias may be introduced.

## Conclusion and recommendations

### Conclusion

The overall incidence rate of DKA in the study area was relatively high. Being an urban dweller, having no family history of DM, having an infection at baseline, having any comorbidities, and having poor glycemic control were found to be the determinants of DKA among adult people with diabetes.

### Recommendations

Effective control measures (a new initiative) should be designed for at-risk populations, particularly people with diabetes who have infection and comorbidity at baseline and who have poor glycemic control. Provide close follow-up for adults' diagnoses of DM on follow up with comorbidity, poor glycemic control, and infection at baseline. To offset missed important variables, it is better to apply a prospective cohort study.

### Supplementary Information

**Additional file 1.** Summary of model comparison based on log likelihood, AIC, and BIC of adult people with diabetes in Woldiya Comprehensive Specialized Hospital from January 1, 2016 to January 1.

**Additional file 2.** Cox-Snell residual test obtained by fitting a Weibull model for adult people with diabetes in Woldiya Comprehensive Specialized Hospital from January 1, 2016 to January 1, 2021

**Additional file 3.** Kaplan-Meier survival curve of adult people with diabetes based on comorbidity in Woldiya Comprehensive Specialized Hospital from January 1, 2016 to January 1, 2021

**Additional file 4.** Kaplan –Meier survival curve of adult people with diabetes based on glycemic control in WCSH from Jan. 1, 2016 to Jan. 1, 2021

**Additional file 5.** Summary of sample size calculation for determinants of Diabetic Keto Acidosis among adult Diabetic patients in Woldiya General Comprehensive Hospital, Northeast Ethiopia, 2021

## Data Availability

The data set used for this study is available from the corresponding author upon reasonable request.

## Abbreviations

AHR: Adjusted Hazard Ratio
CBHI: Community Based Health Insurance
CHR: Crude Hazard Ratio
COPD: Chronic Obstructive Pulmonary Disease
DKA: Diabetic Ketoacidosis
DM: Diabetes Mellitus
FBS: Fast blood sugar
IQR: Inter Quartile Range
NCDs: Non-Communicable Diseases
OHA: Oral hypoglycemic agents
OPD: Out Patient Department
PYs: Person -Years
STATA: Statistics and Data
T_1_D: Type 1 Diabetes Mellitus
T_2_D: Type 2 Diabetes Mellitus
WCSH: Woldia Comprehensive Specialized Hospital
AIC: Akakian information criterion
