# Supplementary material for: Incidence and determinants of diabetic ketoacidosis among people with diabetes in Woldiya comprehensive specialized hospital, Ethiopia: a retrospective cohort study
Source: BMC Endocr Disord. 2024 Mar 11;24:34. doi: 10.1186/s12902-024-01552-1 (PMC10926650; doi:10.1186/s12902-024-01552-1)
Supplement: Supplementary file 1 — Additional file 1. Summary of model comparison based on log likelihood, AIC, and BIC of adult people with diabetes in Woldiya Comprehensive Specialized Hospital from January 1, 2016 to January 1. [file 12902_2024_1552_MOESM1_ESM.docx]

Additional file 1: Summary of model comparison based on log likelihood, AIC, and BIC of adult people with diabetes in Woldiya Comprehensive Specialized Hospital from January 1, 2016 to January 1.

| Model | Log likelihood | Df | AIC | BIC |
| --- | --- | --- | --- | --- |
| Cox regression  Gompertz  Weibull  Exponential  Lognormal  Log logistic | -504.0315  222.1335  223.5375  -163.2176  218.1239  218.2821 | 27  30  30  29  30  30 | 1062.063  -384.2671  -387.0749  384.4352  -386.2478  -386.5641 | 1169.149  -265.2827  -268.0905  499.4535  -267.2634  -267.5797 |

*Df: Degree of freedom AIC: Akakian information criteria BIC: Bayesian information criteria*
