# Supplementary material for: Incidence and determinants of diabetic ketoacidosis among people with diabetes in Woldiya comprehensive specialized hospital, Ethiopia: a retrospective cohort study
Source: BMC Endocr Disord. 2024 Mar 11;24:34. doi: 10.1186/s12902-024-01552-1 (PMC10926650; doi:10.1186/s12902-024-01552-1)
Supplement: Supplementary file 2 — Additional file 2. Cox-Snell residual test obtained by fitting a Weibull model for adult people with diabetes in Woldiya Comprehensive Specialized Hospital from January 1, 2016 to January 1, 2021 [file 12902_2024_1552_MOESM2_ESM.docx]

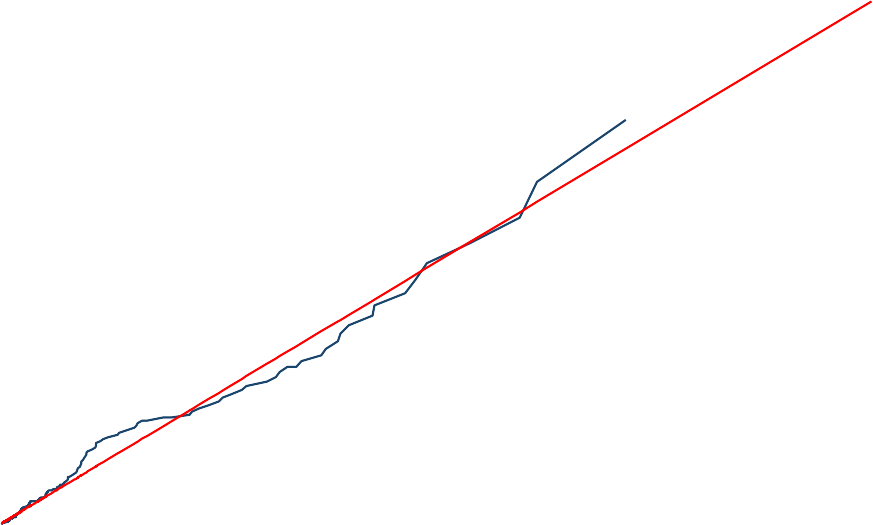


0

2

4

6

Cox-Snell residual

Nelson-Aalen cumulative hazard Cox-Snell residual

2

4

6

Additional file 2: Cox-Snell residual test obtained by fitting a Weibull model for adult people with diabetes in Woldiya Comprehensive Specialized Hospital from January 1, 2016 to January 1, 2021

0
