# Supplementary material for: Incidence and determinants of diabetic ketoacidosis among people with diabetes in Woldiya comprehensive specialized hospital, Ethiopia: a retrospective cohort study
Source: BMC Endocr Disord. 2024 Mar 11;24:34. doi: 10.1186/s12902-024-01552-1 (PMC10926650; doi:10.1186/s12902-024-01552-1)
Supplement: Supplementary file 4 — Additional file 4. Kaplan –Meier survival curve of adult people with diabetes based on glycemic control in WCSH from Jan. 1, 2016 to Jan. 1, 2021 [file 12902_2024_1552_MOESM4_ESM.docx]

Kaplan-Meier survival estimates

0

10

20

30

40

50

Follow up time in month

Good glycemic control poor glycemic control

0.00

0.25

0.50

0.75

1.00

Additional File 4: Kaplan –Meier survival curve of adult people with diabetes based on glycemic control in WCSH from Jan. 1, 2016 to Jan. 1, 2021
