# Supplementary material for: Incidence and determinants of diabetic ketoacidosis among people with diabetes in Woldiya comprehensive specialized hospital, Ethiopia: a retrospective cohort study
Source: BMC Endocr Disord. 2024 Mar 11;24:34. doi: 10.1186/s12902-024-01552-1 (PMC10926650; doi:10.1186/s12902-024-01552-1)
Supplement: Supplementary file 5 — Additional file 5. Summary of sample size calculation for determinants of Diabetic Keto Acidosis among adult Diabetic patients in Woldiya General Comprehensive Hospital, Northeast Ethiopia, 2021 [file 12902_2024_1552_MOESM5_ESM.docx]

Additional file 5:**-** Summary of sample size calculation for determinants of Diabetic Keto Acidosis among adult Diabetic patients in Woldiya General Comprehensive Hospital, Northeast Ethiopia, 2021

| Variables | Assumption | Relative risk | Sample size | 10%  contingency | Reference |
| --- | --- | --- | --- | --- | --- |
| younger age | P1=47.67  p2=32.85 | 1.45 | 368 | 405 | (34) |
| Presence of comorbidity | P_1_=31.94  P_2_=17.19 | 1.86 | 292 | 322 | (27) |
| Presence of infection | P_1_=35.41  P_2_ =14.1 | 2.51 | 146 | 160 | (27) |
